# Parkinsonism Associated with Pathological 123I-FP-CIT SPECT (DaTSCAN) Results as the Initial Manifestation of Sporadic Creutzfeldt-Jakob Disease

**DOI:** 10.1155/2018/5157275

**Published:** 2018-05-31

**Authors:** Sira Carrasco García de León, Juan Pablo Cabello, Ramón Ortiz, Julia Vaamonde

**Affiliations:** ^1^Department of Neurology, University General Hospital, Ciudad Real, Spain; ^2^Intensive Care Unit, University General Hospital, Ciudad Real, Spain

## Abstract

Sporadic Creutzfeldt-Jakob disease (sCJD) is a type of progressive, subacute encephalopathy associated with spongiform degeneration of the central nervous system. sCJD includes a broad and heterogeneous spectrum of clinical variants, but extrapyramidal symptoms and signs at disease onset were rarely reported. We describe a case of unilateral parkinsonism associated with pathological ^123^I-ioflupane SPECT (DaTSCAN) results as the initial manifestation of M129V subtype sCJD patient. To the best of our knowledge, only 2 cases of Creutzfeldt-Jakob disease demonstrating nigrostriatal dopaminergic deficits in vivo using DaTSCAN have been published in the literature.

## 1. Introduction

Parkinsonism as the first symptom of sporadic Creutzfeldt-Jakob disease (sCJD) is rarely described [1–3]. The anatomical involvement of subcortical structures, mainly caudate and putamen, characteristic of this disease, could explain the mechanism of parkinsonism [4]. The involvement of these noncortical areas in CJD has been shown by MRI. However, in vivo measures of neurotransmission in CJD are exceptional. Only presynaptic dopaminergic depletions have been reported in one case each of sCJD and variant CJD by dopamine transporter (DAT) single-photon emission computed tomography (SPECT) [5, 6].

We present the case of a patient with sCJD manifesting with gait abnormality and right-side bradykinesia and hypokinesia, associated with presynaptic dopaminergic deficits. Our case supports the hypothesis of nigrostriatal pathway alterations in CJD.

## 2. Case Presentation

**Figure 1:**
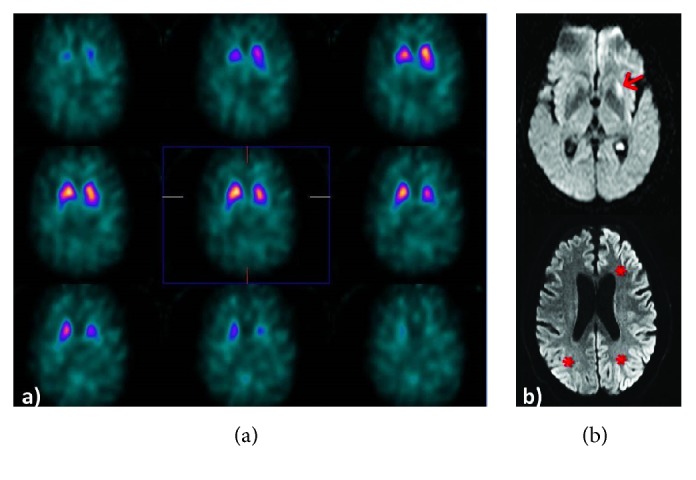
(a) DaTSCAN detected a slight decrease in tracer uptake in the left caudate nucleus, as well as reduced uptake in the putamina (more marked in the left). (b) Brain MRI scan (axial, diffusion-weighted sequences). Above: hyperintensity in left putamen (arrow). Below: disease progression was associated with involvement of the cortex of the left hemisphere and, to a lesser extent, the right temporoparietal cortex (asterisks). Images were taken at a one-month interval.

A 65-year-old man with no relevant family or personal history was admitted due to a 2-day history of mild dysarthria and naming impairment with difficulty finding words and holding a conversation. Furthermore, his family reported at least one-month history of motor awkwardness and gait instability. During the neurological examination, the patient was well oriented, displayed fluent, coherent spontaneous language, and had mild dysnomia. He had remarkable clinical symptoms of parkinsonism: glabellar reflex was persistent; the patient presented a decreased blinking rate, hypomimia, moderate bradykinesia and hypokinesia in the right limbs, and slow gait with reduced right arm swing (Hoehn & Yahr stage I). The examination revealed no further abnormalities. With the clinical suspicion of parkinsonism, we performed a complete blood test performed at baseline, with either normal or negative. A brain MRI scan revealed increased signal intensity in the left putamen with diffusion restriction, suggesting lacunar infarction (Figure 1). By the initial findings obtained in the MRI, vascular study was completed with echocardiogram, Holter monitoring, and Doppler ultrasound of the supra-aortic trunks which showed no significant alterations. Parkinsonism was studied using ^123^I-ioflupane SPECT (DaTSCAN), which revealed asymmetrical tracer uptake in the caudate nuclei (decreased uptake in the left caudate nucleus) and a near-complete lack of activity in the putamina, particularly on the left side (Figure 1). On discharge, the initial working diagnosis was ischemic stroke and idiopathic Parkinson's disease. The patient remained at home. Progression was poor: dysphasia persisted and mobility decreased dramatically until the patient was unable to walk or even hold a standing position. He was readmitted 2 weeks later in a state of stupor. An emergency EEG revealed short-interval (<2 s) lateralized interictal epileptiform discharges in the left hemisphere (triphasic sharp waves and spikes); discharges were of greater amplitude in anterior regions and occasionally spread to the contralateral hemisphere. Within 24 hours, the patient went into coma (Glasgow Coma Scale 3) and was admitted to the intensive care unit. In the following days, and despite administration of several antiepileptic drugs (levetiracetam, valproate, propofol, and midazolam), he remained comatose and displayed clonic movements in the right arm. An additional brain MRI scan performed a month after the initial scan revealed progression of the lesions, with a patchy, gyriform hyperintensity predominantly affecting the cortex of the left hemisphere and the right temporoparietal cortex, visible in diffusion sequences only (Figure 1).

**Figure 2:**
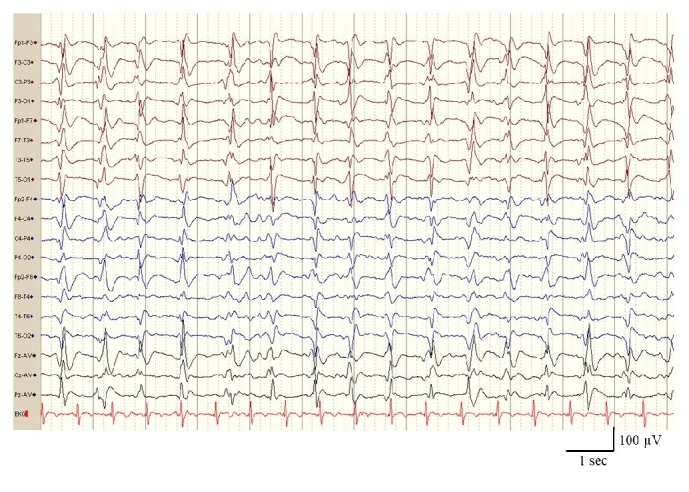
EEG recording showing typical generalized periodic triphasic sharp-wave complexes (PSWCs) occurring at a rate of 1/s, fulfilling EEG criteria of sCJD (20 days before death).

Our patient's rapid neurological deterioration, the presence of myoclonus, and MRI findings pointed to a diagnosis of Creutzfeldt-Jakob disease. To rule out other possible causes of rapidly progressive dementia, we conducted a tumour extension study including tumour markers, onconeural antibodies, and a thoracic-abdominal CT scan; the study yielded negative results. The results from a CSF cell count and a biochemical and microbiological analysis were negative; Harrington test for the determination of 14-3-3 protein on CSF was positive (test was performed at Spain's National Microbiology Centre). An additional EEG performed 2 months after the first EEG revealed disorganised slow background activity, with generalized periodic sharp-wave complexes (PSWCs) at a frequency of 1 Hz (Figure 2). A genetic study found no mutations in the* PRNP* gene; the study of codon 129 polymorphisms revealed methionine/valine heterozygosity (M129V). The probable diagnosis of sporadic CJD was reached based on the World Health Organization (WHO) diagnostic criteria [7]. He died 5 months after disease onset; autopsy was not granted.

## 3. Discussion

M129V heterozygote subtype sCJD, as our case, is characterized by mean age at onset of symptoms about 65 years, average clinical duration 4 months, and a spectrum of signs at onset that include cognitive decline, ataxia, language dysfunction, visual signs of central origin, and myoclonus [2]. Diffusion-weighted imaging hyperintensity in the basal ganglia in about 70% of cases and PSWCs are recorded in EEG in about 80% [2]; the same applies in our case. The rarity of our patient is an unusual parkinsonism as sign at onset, only described in one-fourth of M129V subtype sCJD patients [2].

Further, lesions to the nigrostriatal pathway have never been studied in detail [8]. To our knowledge, only 2 cases of Creutzfeldt-Jakob disease demonstrating nigrostriatal dopaminergic deficits in vivo using DaTSCAN have been published in the literature [5, 6]. This technique uses ^123^I-ioflupane or ^123^I-FP-CIT, a tracer whose chemical name is N-*ω*-fluoropropyl-2*β*-carbomethoxy-3*β*-(4-[^123^I]iodophenyl) nortropane. This cocaine analogue has a high binding affinity for and acceptable sensitivity to dopamine transporters (DAT), making it an excellent marker of viability and function of nigrostriatal neurons. Numerous studies of Parkinson's disease have evaluated the use of this technique to confirm clinical diagnosis. In the context of Creutzfeldt-Jakob disease, in contrast, DaTSCAN may be useful for in vivo confirmation of the presence of striatal dopaminergic degeneration.

Although our patient was admitted due to pseudoictal symptoms of language impairment, his gait disorder had appeared a month previously. In our case, the DaTSCAN revealed abnormal tracer uptake in the left caudate nucleus and reduced tracer uptake in the putamina (especially in the left putamen); these findings were coherent with the patient's symptoms of right-sided bradykinesia. It may be argued, however, that the patient had mixed parkinsonism (abnormal structural imaging and neurodegenerative parkinsonism) based on functional neuroimaging results and the presence of a lesion to the left putamen on diffusion-weighted MRI sequences; this lesion may reflect the spongiform changes typical of sCJD. These findings were consistent with anatomical pathology results from the postmortem studies, which revealed dopaminergic neuron loss in the substantia nigra, caudate nucleus, and putamen [8]. This supports the hypothesis of simultaneous presynaptic and postsynaptic degeneration of the nigrostriatal pathway in Creutzfeldt-Jakob disease [4, 8].

## 4. Conclusion

In conclusion, parkinsonism in CJD may be caused by lesions to the basal ganglia affecting the lenticular nucleus, or by nigrostriatal pathway degeneration. Much remains to be understood about DaTSCAN as an in vivo functional technique for the study of basal ganglia motor circuits and their role in the pathogenesis of this symptom in CJD. In any case, DaTSCAN seems to be a promising, noninvasive method for identifying lesions to the dopaminergic pathway; combined with MRI, it may help in the diagnosis of CJD.
